# Clinical Diathermy Performance Evaluation of Multi-hour Sustained Acoustic Medicine Treatment with 2.5% Diclofenac Ultrasound Coupling Patch

**Published:** 2023-06-20

**Authors:** Tabitha F. Hendren, Natalie R. Yeretzian, Kiranmayee Bavanasi

**Affiliations:** 1 Department of Engineering, ZetrOZ Systems LLC, Trumbull, CT USA;; 2 Department of Biomedical Engineering, University of Cincinnati, Cincinnati, OH USA

**Keywords:** Ultrasound, Diclofenac, Diathermy, Acoustic impedance, Sustained acoustic medicine

## Abstract

**Background:**

Low-intensity Continuous Ultrasound (LICUS) therapy heals soft tissue injuries. It alleviates acute and chronic musculoskeletal pain by activating multiple healing processes through its diathermic and mechanotransducive properties. Diclofenac has been FDA-approved as a Non-Steroidal Anti-Inflammatory Drug (NSAID). It is an analgesic and anti-inflammatory drug available in oral and topical forms. Adding 2.5% diclofenac sodium to ultrasound coupling gel can be used to deliver LICUS in addition to the analgesic effects of diclofenac sodium without altering the diathermic and acoustic effects of the ultrasound penetration with no undesired adverse effects.

**Objective:**

To determine the effects of adding 2.5% diclofenac sodium to standard aqueous ultrasound gel on the ultrasound coupling and diathermic properties of a long duration Sustained Acoustic Medicine (SAM) treatment.

**Methods:**

In a two-phase study, first, the acoustic and diathermic changes were determined in bovine tissue during 4-hour-long SAM stimulation at 1 cm, 2 cm, and 5 cm with aqueous and 2.5% diclofenac ultrasound coupling patch. Then, in the second phase, the heating profiles were recorded with and without 2.5% diclofenac gel in 54 healthy adult subjects at the forearm and calf during the SAM treatment.

**Result:**

The addition of 2.5% diclofenac sodium significantly increased coupling gel density, acoustic impedance, and signal propagation (p<0.0001) with little or no effect on the diathermic profiles at 1 cm, 2 cm, and 5 cm depth. The coupling gel with 2.5% diclofenac sodium sustained the therapeutic ultrasound intensity longer than the aqueous coupling gel (5.5 cm relative to 4.5, p<0.0009). No significant diathermic difference was recorded on the calf and forearm skin with a 2.5% diclofenac ultrasound gel coupling patch.

**Conclusion:**

Adding 2.5% diclofenac sodium to ultrasound gel increases acoustic impedance, improves ultrasound signal coupling into deep tissue, and provides longer sustained deep tissue heating without negatively impacting the diathermic profile during SAM treatment.

## INTRODUCTION

Low-Intensity Continuous Ultrasound (LICUS) therapy is increasingly used to heal soft tissue injuries and relieve pain in acute and chronic musculoskeletal ailments [1–4]. Ultrasound penetrates the damaged tissue to enhance the healing process by several mechanisms, including diathermy and mechanotransduction [5–8]. The localized increase in the temperature, cellular migration, and biomolecular activity enhances circulation, vasodilation, oxygenation, and nutrient exchange, boosting cellular metabolism and tissue regeneration [6–11]. The LICUS is transmitted at frequencies between 1 and 3 MHz at intensities of less than 0.5 W/cm^2^ for 10 minutes to 4 hours [4,12]. The consistent, soothing, and vigorous heat penetrates deep into connective tissue and muscles with little to no adverse effects on the tissue [9,13]. The application of low-intensity ultrasound between 2–20 MHz is also used in clinical diagnostic and imaging purposes, depending on the application and target tissue. The diagnostic and imaging use of ultrasound relies on the difference in acoustic impedance of tissues. High-Intensity Focused Ultrasound (HIFUS) uses > 10 W/cm^2^ intensity, generating localized heating, and is more commonly commonly applied in targeting solid tumors. The HIFUS can be used as an alternative to invasive surgery [14]. Diclofenac Sodium is a broadly used Non-Steroidal Anti-Inflammatory Drug (NSAID). It is an analgesic, anti-inflammatory, and antipyretic agent [15]. It acts by inhibiting cyclooxygenase-1 and 2, regulating the production of prostaglandin and various pro-inflammatory biomarkers, including Tumor Factor alpha (TNFα), Interleukin-6 (IL-6), and Interleukin-8 (IL-8) [16]. Topical diclofenac has been shown to be efficacious in osteoarthritis, rheumatic arthritis, and soft tissue pain injuries such as sprains, ligament partial or total rupture, and contusions [16]. Oral diclofenac has been shown to have adverse vascular and gastrointestinal effects, and the efficacy of topical diclofenac is limited due to limited penetration through the skin tissue [15,17,18]. The ultrasound acoustic waves are generated by a piezoelectric element and require a dense medium to travel. Viscous ultrasound gel facilitates acoustic wave transmission from the transducer to the target tissue. The presence of the dense impedance-matched medium allows the transmission of acoustic vibration to the tissue with little or no loss of acoustic energy and intensity. In addition, the localized acoustic force can provide essential mechanical and thermal stimulation to increase profusion, porosity, and cellular activity [19,20], thereby synchronizing ultrasound’s mechano-transduction and diathermic properties along with topical NSAID application benefits [19,21,22]. The United States Food and Drug Administration (FDA) has cleared Sustained Acoustic Medicine (SAM) for treating musculoskeletal injuries. SAM provides low-intensity continuous ultrasound at 1.3 W and 0.132 W/cm^2^, which equates to about 5,000 Joules per hour or just under 20,000 Joules for a typical 4-hour treatment session [10,21,23]. The meta-analysis by Winkler, et al. demonstrated the clinical efficacy of SAM in numerous musculoskeletal disorders, including myofascial pain, rotator cuff tendinopathy, knee osteoarthritis, and upper shoulder and neck pain [23]. In addition, Uddin, et al. has elaborated on the underlying mechanism of LICUS and SAM in multiple pathologies such as traumatic bone fracture, osteoarthritis, thrombosis, and pain management [6].

The use of SAM with diclofenac ultrasound gel has been reported by Madzia et al. The SAM treatment with 1% topical diclofenac significantly alleviated the osteoarthritis-induced pain and improved Western Ontario McMaster Outcome (WOMAC) scores after 7 days of treatment [24]. The treatment efficacy and improved outcomes were attributed to the diathermic and mechanotransducive abilities of the SAM treatment over a 4-hour time. Jarit, et al. presented the use of SAM with 2.5% diclofenac in the treatment of musculoskeletal injuries that failed to respond to physical therapy [25]. After 4-weeks of daily home use, patients reported a significant pain reduction, global health, and functional improvement with SAM treatment. This study aims to determine the ultrasound coupling and diathermic effects of adding 2.5% diclofenac sodium to the ultrasound coupling patch in the SAM treatment system.

## MATERIALS AND METHODS

### Instrumentation

The FDA-cleared SAM device (model sam-12, ZetrOZ Systems LLC., Trumbull, CT) consists of a pair of small rechargeable wireless transducer heads, clipping onto an adhesive patch pre-loaded with either standard coupling ultrasound gel or 2.5 w/v diclofenac coupling patch. Adhesive patches are directly applied to the skin above the treatment area. The SAM device provides continuous low-intensity continuous ultrasound at 0.132 W/cm^2^ for a 4-hour treatment delivering 18,720 at 3 MHz with 0.65 W of power from two transducer heads each with a 5cm^2 radius treatment area.

### Laboratory methods

#### Intensity measurement:

The therapeutic intensity in bovine tissue at different depths was measured using a hydrophone with both coupling gels. A 3 MHz, 4-cycle, sinusoidal signal was generated from a Tektronix function generator (model AFG3021B) and ran through a SAM piezoelectric transducer creating a simulated ultrasound signal similar to the sam-12 device. An ONDA Hydrophone (model HNR-1000) was used to measure and record the signal output at different depth levels using a Tektronix oscilloscope (model TDS2014B).

The bovine tissue was sliced into 1 cm, 2 cm, 3 cm, 4 cm, and 5 cm thickness, 5 cm diameter discs, with 6 samples per measurement. The bovine tissue was placed between an active transducer and the hydrophone and coupled to both pieces using redundant ultrasound gel. The signal was recorded *via* the hydrophone and input to an oscilloscope, where the intensity was displayed and recorded. The intensity measurements were recorded with aqueous coupling gel and with 2.5% diclofenac coupling gel. The data was displayed on a graph found in the results section below. The effect of 2.5% diclofenac on the speed of sound and acoustic impedance was also quantified using a similar experimental setup without the bovine tissue, as shown in Figures 1A and 1B. The hydrophone was held at a stable, constant distance from the piezo transducer face outputting a consistent ultrasound signal by a preprogrammed oscilloscope. Ultrasound gel was inserted between the transducer face and the hydrophone tip. The signal was generated and recorded by the same oscilloscope at two different independent channels concurrently, and the time delay was recorded. The speed of sound was calculated using Equation (1) and the experimental setup in Figures 1A and 1B (Figures 1A and 1B).


(1) $$c=\frac{d}{t}$$

$$\text{speed of sound}=\frac{\text{ distance }(\text{meters})}{\text{ time delay }(\text{seconds})}$$

speed of sound=(distance(meters))/(time delay(seconds))

The speed of sound was used to calculate the acoustic impedance based on density determined with a mass and volume calculation using Equation (2). The acoustic impedance was found for both coupling gels, with and without 2.5% diclofenac gel.


(2) Z=ρ*c

$$\text{Acoustic Impendance}=\text{density }\left(\frac{kg}{m^{3}}\right)*\text{speed of sound }\left(\frac{m}{s}\right)$$

#### Bovine diathermy:

Bovine muscle tissue, 800 mg ± 200 mg, bought from a local butcher, was normalized to room temperature and pressure. The ultrasound coupling gel (3 ml) with and without 2.5% diclofenac was placed on the surface of the tissue and well-secured using bandages to prevent any leakage of the gel. The SAM transducer was clipped into the plastic coupling ring that is sealed to the bandage (Figure 1C). Stainless steel thermocouple probes were stereotactically inserted 2.5 inches into the bovine muscle tissue directly under the center of the transducer. Three probes were stereotactically inserted at 1 cm, 2 cm, and 5 cm depths (Figure 1D). The internal temperatures were continuously monitored during the treatment using OMEGA thermocouple probes (TJ36-ICSS-116G-6-GG) and recorded on a DAQPRO Data logger (OM-DAQPRO-5300). The data logger was set to continuously collect thermocouple K values every ten seconds until the logger was stopped. The tissue was stimulated for 4 hours by the SAM device (Figure 1C and 1D).

### Clinical methods

#### Participants:

Fifty-six individuals were recruited for the study. Fifty-four healthy male and female subjects, aged 18–70 years, were enrolled in the study with IRB-approval from the Advarra IRB based out of Columbia, MD (Pro00060493). The study adhered to the principles outlined in the Declaration of Helsinki and was registered on clinicaltrails.gov (NCT05259995). All subjects signed complete informed consent forms before the start of the study. Pregnant/nursing or subjects with a topical wound in the treatment area were excluded from the study. Subjects were given $100 completion per session. Twenty-six subjects were male, and twenty-eight females between 18–65, averaging 28 years old with 16.4 to 37.3 BMI with an average BMI of 25.8, were selected for the study.

### Procedure

**Figure 2:**
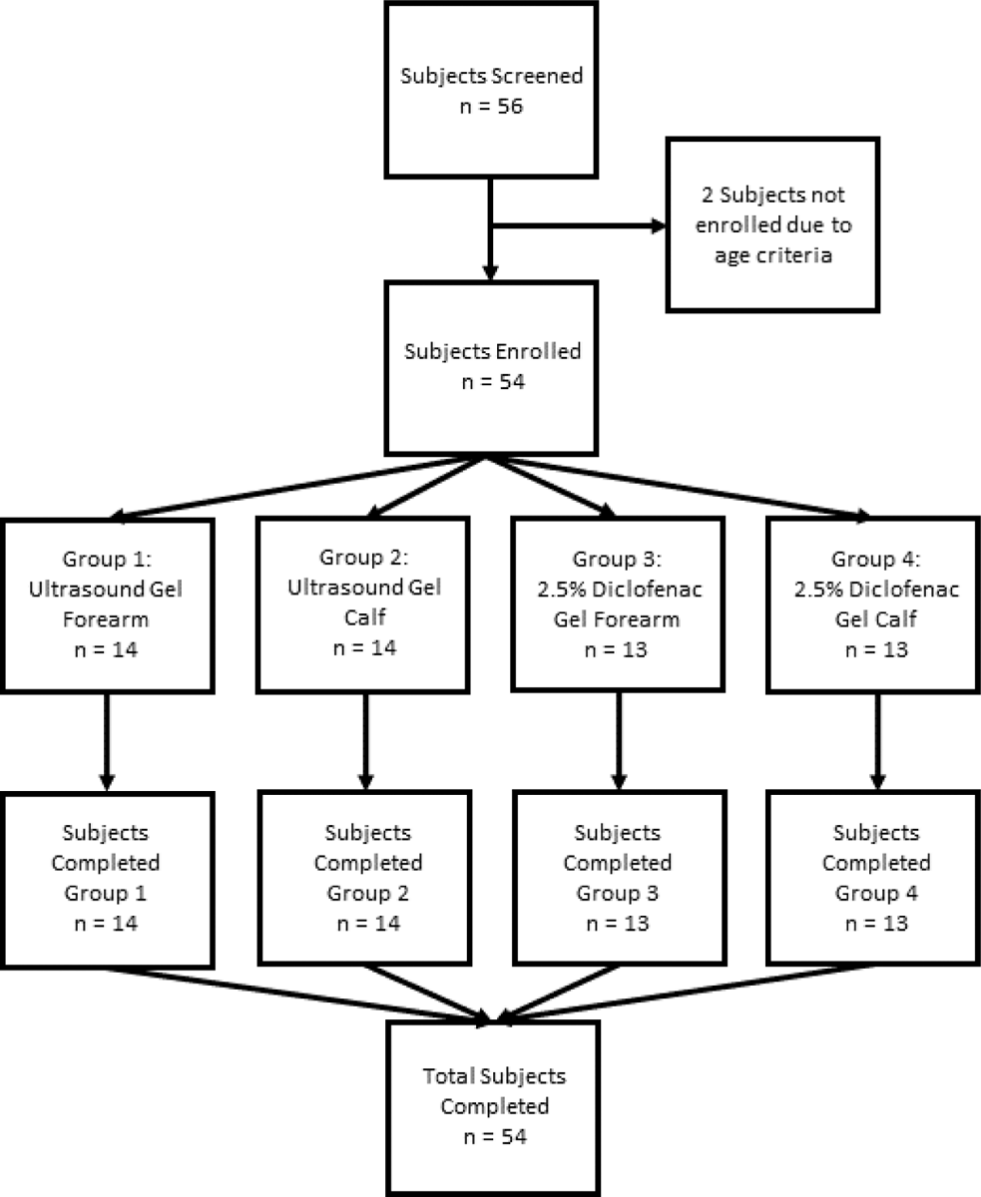
PRISMA flow diagram for screening, enrollment, group assignment, and completion (100%) for participants in clinical study.

**Figure 3:**
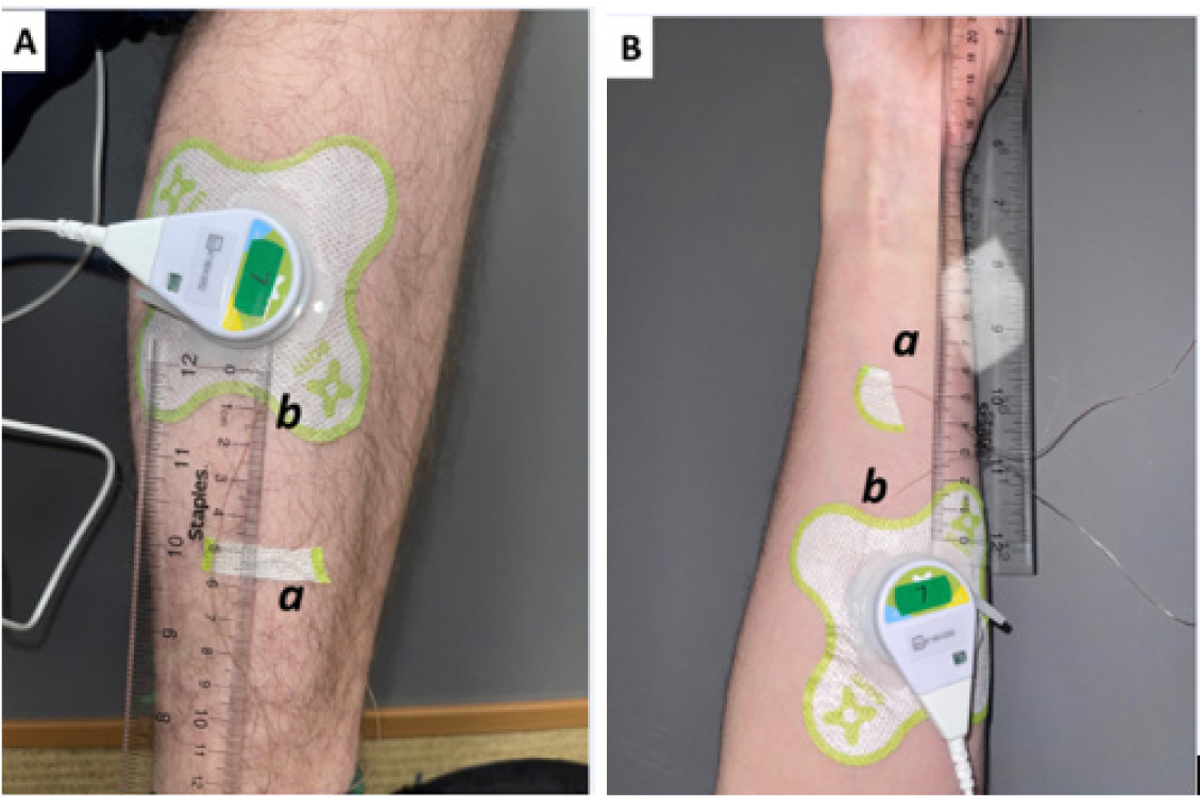
Human subject experimental set-up (A) On the calf and (B) the forearm. **Note:** (a) Untreated skin surface micro thermocouple probe adhered in place with a bandage “(b) treated” b) treated skin surface micro thermocouple probe placed directly underneath the active transducer and held in place with the SAM bandage (ultrasound patch).

Participants were randomly assigned to one of four groups using random number allocation on Microsoft Excel. The study PRISMA flow diagram is shown in Figure 2. Group 1 subjects received SAM ultrasound treatment on the forearm with standard ultrasound coupling gel. Group 2 received the same treatment and standard gel on the calf muscle. Group 3 participants were treated with SAM+2.5% diclofenac ultrasound coupling gel on the forearm. Group 4 was treated with SAM+2.5% diclofenac ultrasound coupling gel on the lowercase calf muscle. Figures 3A and 3B show the transducer positioning on the Calf and forearm, respectively. All the stimulation sessions were comprehensively completed at the research study site with federal-wide assurance. Local temperature readings were recorded for a 4-hour SAM treatment protocol in healthy human volunteers measured with a micro thermocouple probe on the skin surface under the active transducer. The temperature change was recorded at the untreated site as a reference point. Standard aqueous ultrasound coupling gel was used with the transducers in 28 subjects (n=28), and 26 volunteers received treatment using the 2.5% diclofenac ultrasound coupling gel (n=26). Temperatures were recorded at the skin’s surface beneath the active transducer on either the forearm (n=27) or calf muscle (n=27). Control temperatures were recorded at the skin’s surface 6 cm away from the treatment site, as shown in Figures 4A and 4B. Temperatures were measured using an OMEGA micro thermocouple (Model: 5SC-TT-K-40–36) and recorded with the OMEGA DAQPROs, also used in laboratory experiments. The thermocouples adhered to the skin with SAM bandage pieces. The data was uploaded to DAQLAB software on the computer to be analyzed (Figures 4A and 4B).

### Statistical analysis

A statistical analysis was conducted to assess the statistical differences in ultrasonic signal penetration, gel acoustic properties, and temperature performance for the two coupling gels used. T-tests were conducted using sample size, mean, and standard deviation. Statistical difference with p < 0.05 was considered significant difference between measurement groups. In addition, the statistical difference between temperature values of the two coupling gels at the same depths over the 4-hour SAM treatment was evaluated using One-Way ANOVA with Prism GraphPad.

## RESULTS

### Laboratory results

2.5% Diclofenac gel properties: The speed of sound calculated for 2.5% diclofenac ultrasound gel was 1513.24 ± 2.24 m/s. The speed of sound calculated for the current SAM ultrasound gel was 1512.64 ± 2.39 m/s with no significant difference (p=0.69). Acoustic impedance was calculated using the speed of sound and the density of both gels, respectively. Acoustic impedance for 2.5% diclofenac ultrasound gel was 1.599 ± 0.0001 MPa⸱s/m^3^ and 1.543 ± 0.002 MPa⸱s/m^3^ for the SAM ultrasound gel. The acoustic impedance between both coupling gels was statistically different (p<0.0001) (Table 1).

### Bovine performance testing

The SAM device did not fall below the therapeutic ultrasound threshold of bone-growth stimulators (30 mW/cm^2^) until approximately 4.5 cm in bovine tissue depth with standard ultrasound coupling gel and 5.5 cm depth with 2.5% diclofenac ultrasound coupling gel.The calculated intensity values confirm that the 30 mW/cm^2^ threshold was passed between 4.5 cm and 5.5 cm, and the addition of 2.5% diclofenac significantly (p=0.009) enhances ultrasound propagation into tissue by approximately 1 cm. The highest acoustic intensity was recorded at the surface of the transducer, and decayed with increasing depth. The exponential decay rate was delayed with 2.5% diclofenac, showing improved acoustic impedance matching with the 2.5% diclofenac sodium gel (Table 2).

The diathermic analysis at 1 cm, 2 cm, and 5 cm in the bovine tissue showed a distinct diathermic profile at each intramuscular depth and distance from the transducer surface. The highest temperature increase change rate was recorded closest to the transducer surface, 1 cm intramuscular depth. The temperature increased from an average of 20.63°C and peaked at approximately 35.31°C after 240 min of the treatment at 1 cm depth; Δ14.68°C was the highest change in tissue temperature for the first 90 min. The smallest temperature increase was recorded at 5 cm intramuscular depth from 20.36°C to 24.56°C with a total increase of Δ4.20°C and gradually increasing over 240 mins. The temperature significantly increased over 240 min (4 hours stimulation). After the treatment, the temperature decrease time was less. At the end of the SAM treatment, temperature reduced significantly over the first 60 mins, with a relatively slower rate of decrease recorded in the bovine tissue treated with 2.5% diclofenac gel due to the deeper therapeutic acoustic penetration. However, no distinctive diathermic profile differences were recorded between the SAM coupling patches without and with 2.5% diclofenac across all three depths.

### Clinical human subject’s results

The effect of the diclofenac coupling patch on skin temperature was assessed on the adult human calf and forearm. Prior to the start of the treatment, skin temperature at the calf was 28.78°C ± 1.39°C and 29.29°C ± 1.39°C respectively, without and with 2.5% diclofenac coupling gel, and 29.96°C ± 1.69°C and 30.32°C ± 1.64°C respectively without and with 2.5% diclofenac coupling gel on the human forearm. After the 4 hours of the SAM treatment, approximately the same increase in the temperature was recorded at both sites (Figures 4C and 4D). The skin temperature at the calf increased to 40.56°C ± 1.33°C and 41.46°C ± 1.73°C respectively, without and with 2.5% diclofenac coupling gel. On the forearm, skin temperature increased to 40.04°C ± 2.17°C and 40.22°C ± 1.95°C respectively, without and with 2.5% diclofenac coupling gel. The presence of diclofenac did not affect the surface temperature change after the 4-hour treatment. No significant effect was recorded at either site before or after treatment due to the presence of 2.5% diclofenac sodium.

## DISCUSSION

Ultrasound is an acoustic energy source generated from the vibration of piezoelectric element-initiated sound waves. It requires an aqueous medium to travel from one point to another without dissipating significant energy over a long distance [26–28]. The density of the medium plays an essential role in the travel of ultrasound and associated energy. Thus, it is crucial to understand the impact of a change in density on the thermodynamics of ultrasound and its potential impact on the therapeutic effects. The coupling medium must be liquid enough to flow to fill gaps between the vibrating source (piezoelectric element) and the effector (human skin, organ). Diclofenac is a commonly used NSAID in musculoskeletal pain management. It has relatively high COX-2 isoenzymes inhibition relative to COX-1 and PGE2 production *via* monocytes and platelets [15,16,18]. The inhibition of the PGE2 reduces the localized inflammation and pain sensation systematically [29,30]. However, at a lower level, diclofenac also inhibits the COX-1 isoenzymes, which regulate the prostaglandins that protect gastrointestinal mucosa. Thus, the prolonged systemic use of diclofenac disturbs the mucosal lining and results in ulcers and other intestinal and urinary tract complications. The localized, topical application of diclofenac has shown to be effective. However, it is limited due to the limited penetration through the skin tissue, which significantly varies across different skin sites depending on the skin’s depth and porosity. Previous studies have shown that topical application of diclofenac in conjunction with ultrasound stimulation increases the efficacy of treatment. vân Der Biji, et al. has shown that sonification has increased the diclofenac flux rate through human skin specimens [31]. Masterson, et al. showed increased NSAID penetration through skin-like tissues by 3.8-fold when actively stimulated by the SAM device for 4 hours [21]. Bukhari, et al. has shown an improved reduction in swelling after acute injuries at different anatomical sites in human subject’s post-diclofenac sonophoretic treatment [19]. In a multi-center study, Madzia, et al. have shown alleviated pain reduction and improvement in WOMAC score in knee osteoarthritis patients after being treated with SAM + 1% diclofenac hydrogel for 7 days [24]. Jarit, et al. has shown significant injury pain relief in tendon, muscle, and bone injuries with SAM + 2.5% diclofenac gel for 4 weeks [25]. Multiple studies have shown synergistic responses of ultrasound and diclofenac application [19,21,24,32]. This study demonstrates, for the first time, that the addition of diclofenac can potentially enhance the ultrasound acoustic penetration into the biological tissue. The long-duration SAM treatment renders it essential to ensure that additional 2.5% diclofenac sodium would not negatively impact the diathermic properties of SAM treatment and potentially cause undesired adverse effects. The data from this study shows that the addition of 2.5% diclofenac to the gel changes the density of the coupling patch and improves ultrasound coupling into deeper tissue. The diclofenac coupling gel provides similar diathermic heating properties through the 5 cm of the bovine tissue while providing slower cooling and deeper ultrasonic signal propagation. The slower cooling directly results from an overall increase in ultrasound transmission into the bovine muscle tissue (more energy delivered) due to the improved acoustic impedance characteristics of the 2.5% diclofenac ultrasound gel.

This can also be visualized with generally higher diathermy measurements at 2 cm and 5 cm across all time points measured, including the cool-down phase. Human subject testing further demonstrated similar safe and effective heating profiles on the calf and forearm with both gel types.

The study has some limitations as it uses bovine tissue for deep tissue measurements; future studies may use ex vivo human tissue specimens or large porcine animal models to further assess the efficacy of the long-term SAM treatment with 2.5% diclofenac.

## CONCLUSION

The 2.5% diclofenac ultrasound gel showed increased acoustic impedance, improved ultrasound signal coupling into deep tissue, and provided longer sustained deep tissue heating without negatively impacting the diathermic profile during SAM treatment. The 2.5% diclofenac coupling gel may be considered a viable long duration ultrasound coupling agent.

## Figures and Tables

**Figure 1A and 1B:**
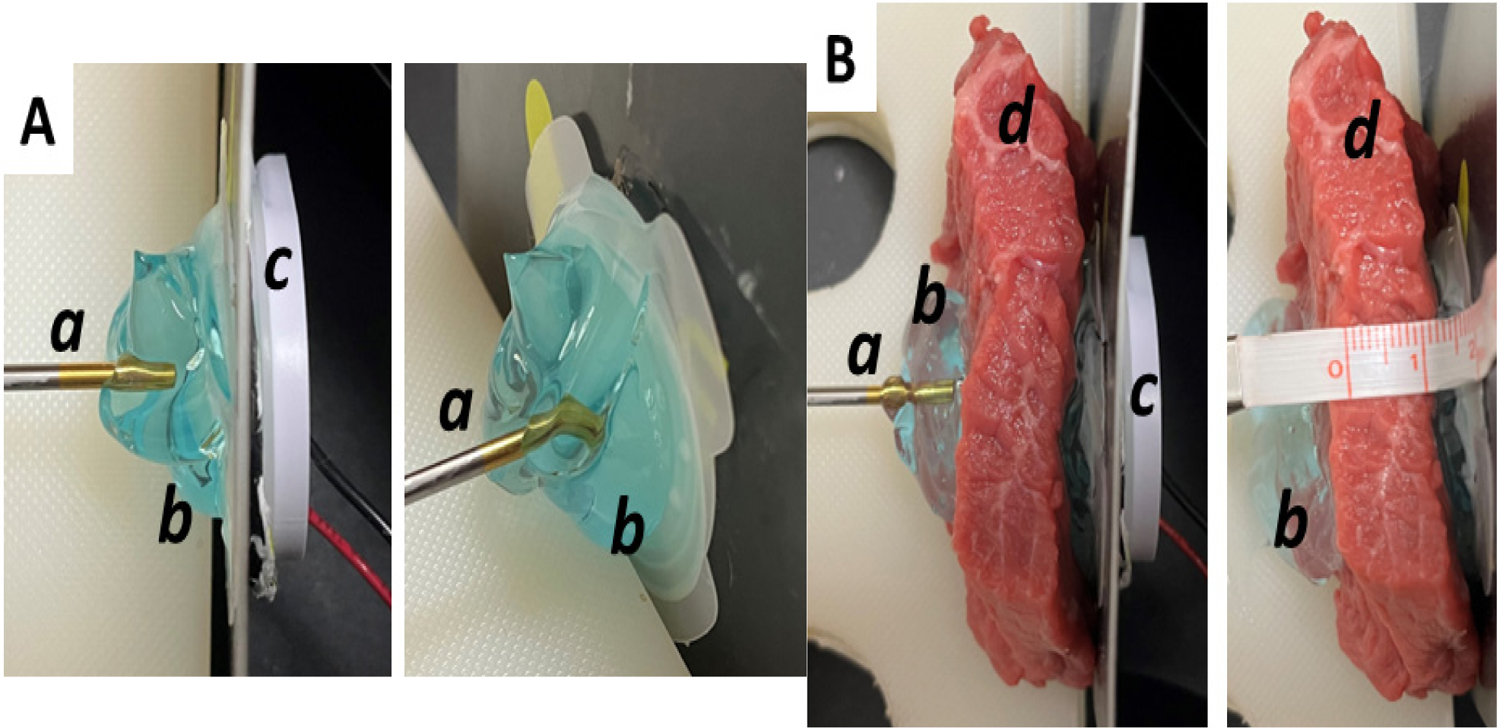
Experimental set-up: For intensity measurement without bovine muscle tissue (1A) and with bovine muscle tissue (1B). **Note:** (a) hydrophone connected to an oscilloscope for readings; (b) standard ultrasound coupling gel; (c) LICUS transducer powered by function generator; (d) bovine muscle tissue cut to be 1 cm thick.

**Figure 1C and 1D:**
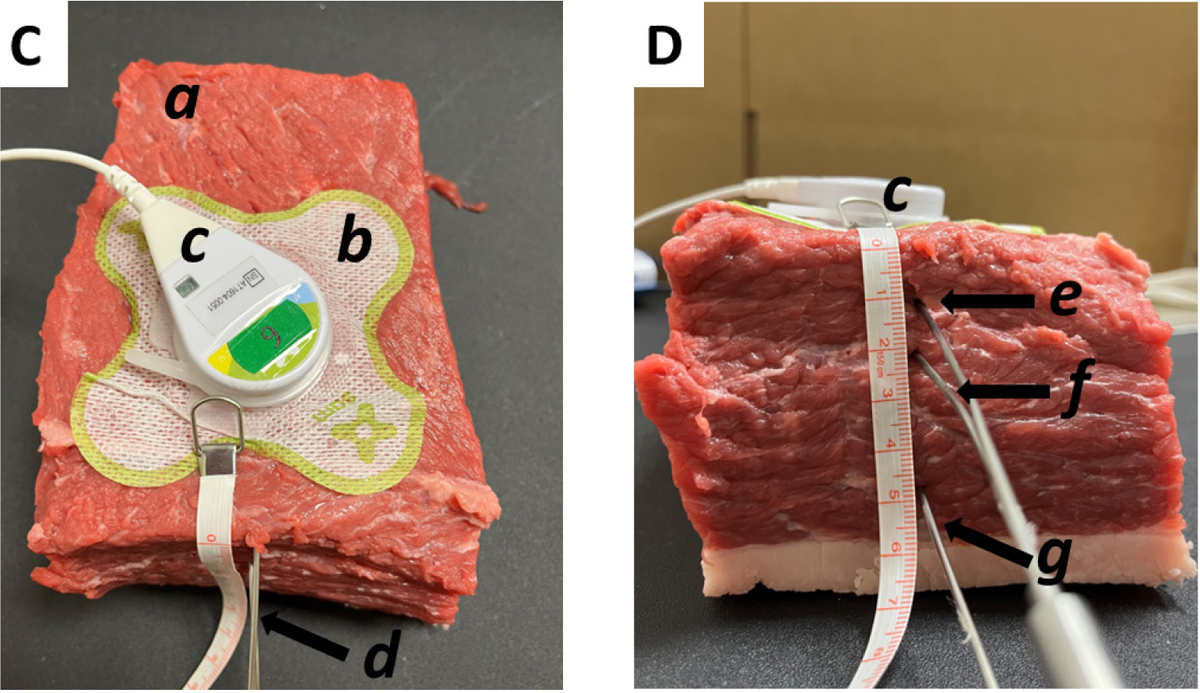
Experimental set-up: Deep tissue diathermy using bovine muscle tissue. The transducer was placed on top (1C). Front view where the thermocouple probes were inserted (1D). Note: (a) Bovine muscle tissue; (b) SAM bandage containing ultrasound coupling gel; (c) SAM transducer to deliver LICUS; (d) OMEGA Thermocouple Probe; (e) Thermocouple probe inserted at 1 cm depth; (f) Thermocouple probe inserted at 2 cm depth; (g) Thermocouple probe inserted at 5 cm depth.

**Figure 4A:**
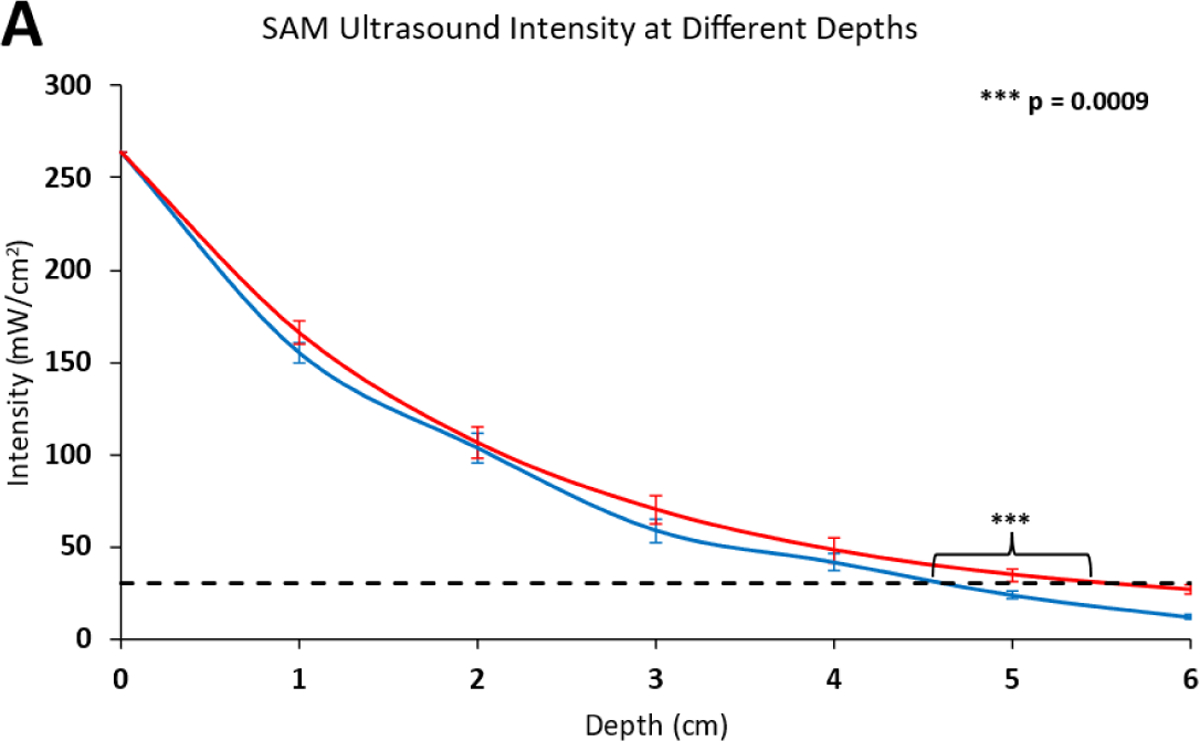
Intensity of SAM ultrasound at increasing depths in bovine muscle tissue compared to the intensity threshold for therapeutic benefits of commercially available ultrasound treatment. **Note:** (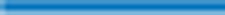):SAM with Standard Gel; (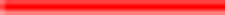): SAM with 2.5% Diclofenac Gel; (––):Therapeutic Ultrasound Treatment Threshold.

**Figure 4B:**
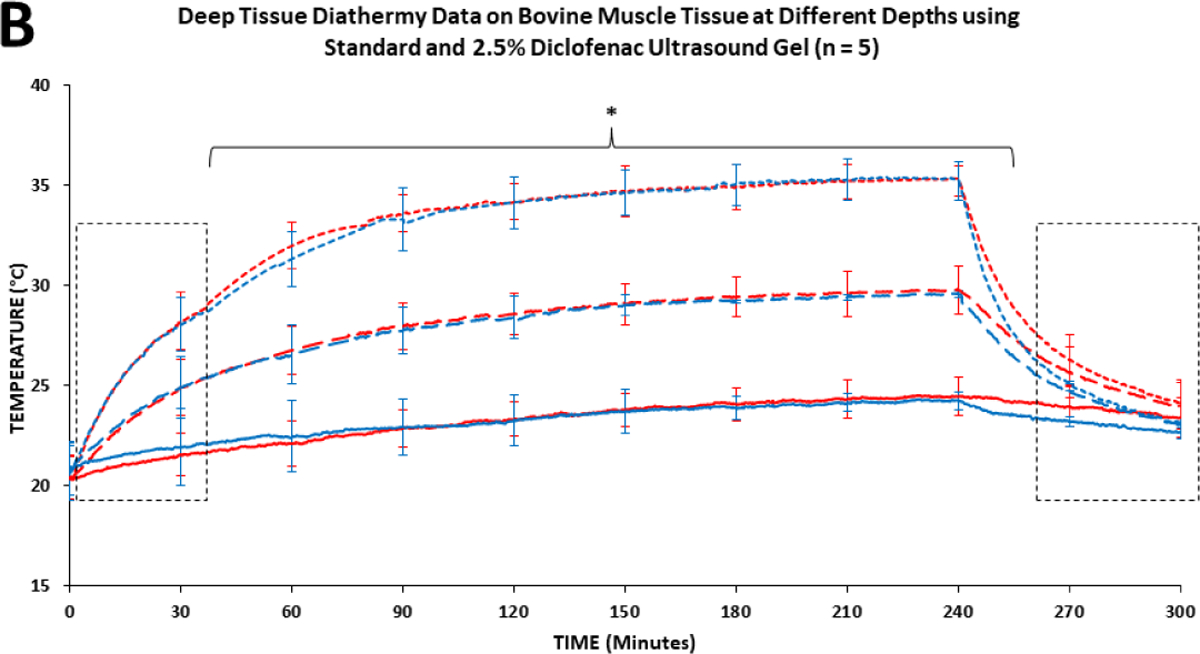
The temperature during SAM stimulation on bovine muscle tissue at various depths(1 cm, 2 cm, 5 cm) using two different coupling patches (with and without 2.5% diclofenac). The diathermic profiles of both coupling gels at each of the varying depths are not significantly different. About 45 minutes into treatment, the temperature values at each depth (1 cm, 2cm, and 5 cm) are statistically different from one another for both coupling gels. **Note:** * = (p < 0.05) **Note:** (–––) 1 CM 2.5% Diclofenac Gel; (––) 2 CM 2.5% Diclofenac Gel; (—) 5 CM 2.5% Diclofenac Gel; (–––) 1 CM Standard Gel; (––) 2 CM Standard Gel; (—) 5 CM Standard Gel.

**Figure 4C:**
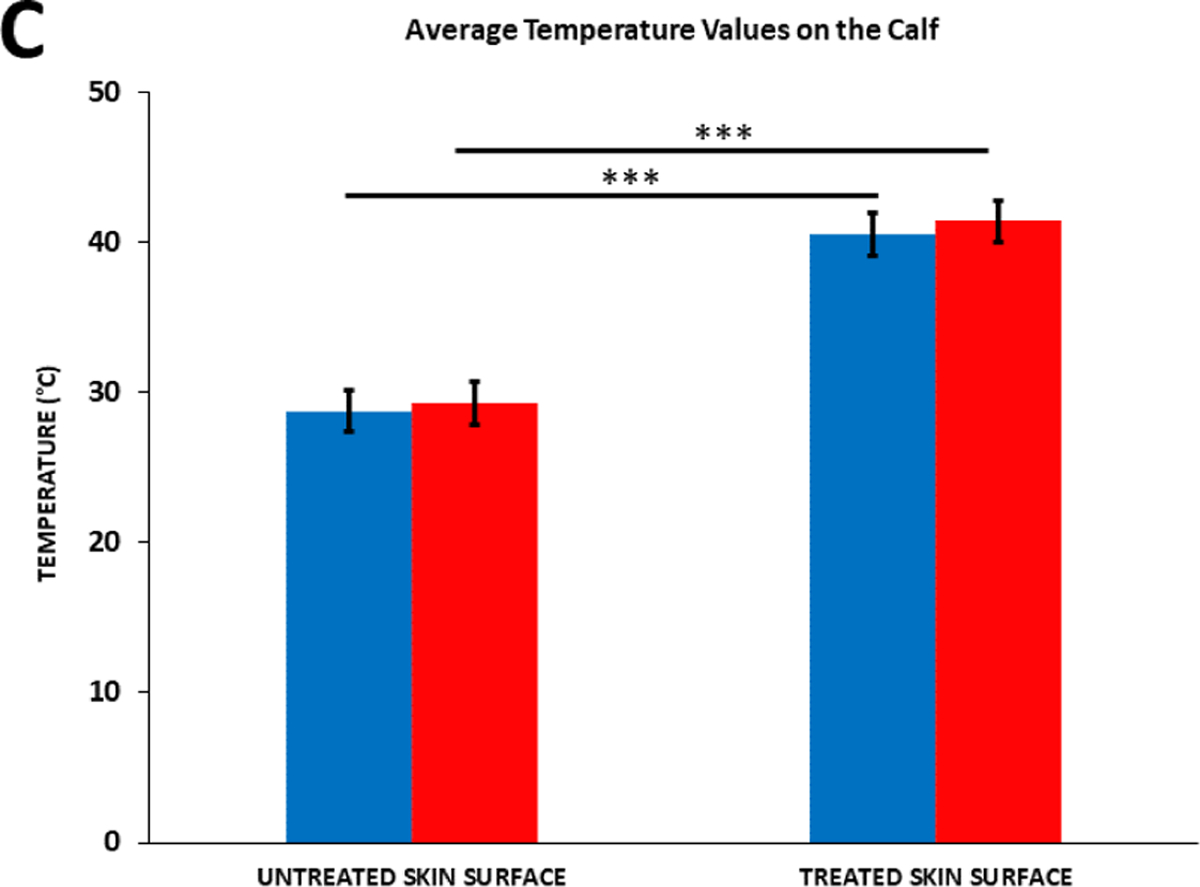
Average temperature values on the calf before and after 4 hours of SAM stimulation. **Note:** (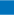) Ultrasound Gel; (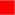) 2.5% Diclofenac Ultrasound Gel **Note:** *** = (p < 0.0001).

**Figure 4D:**
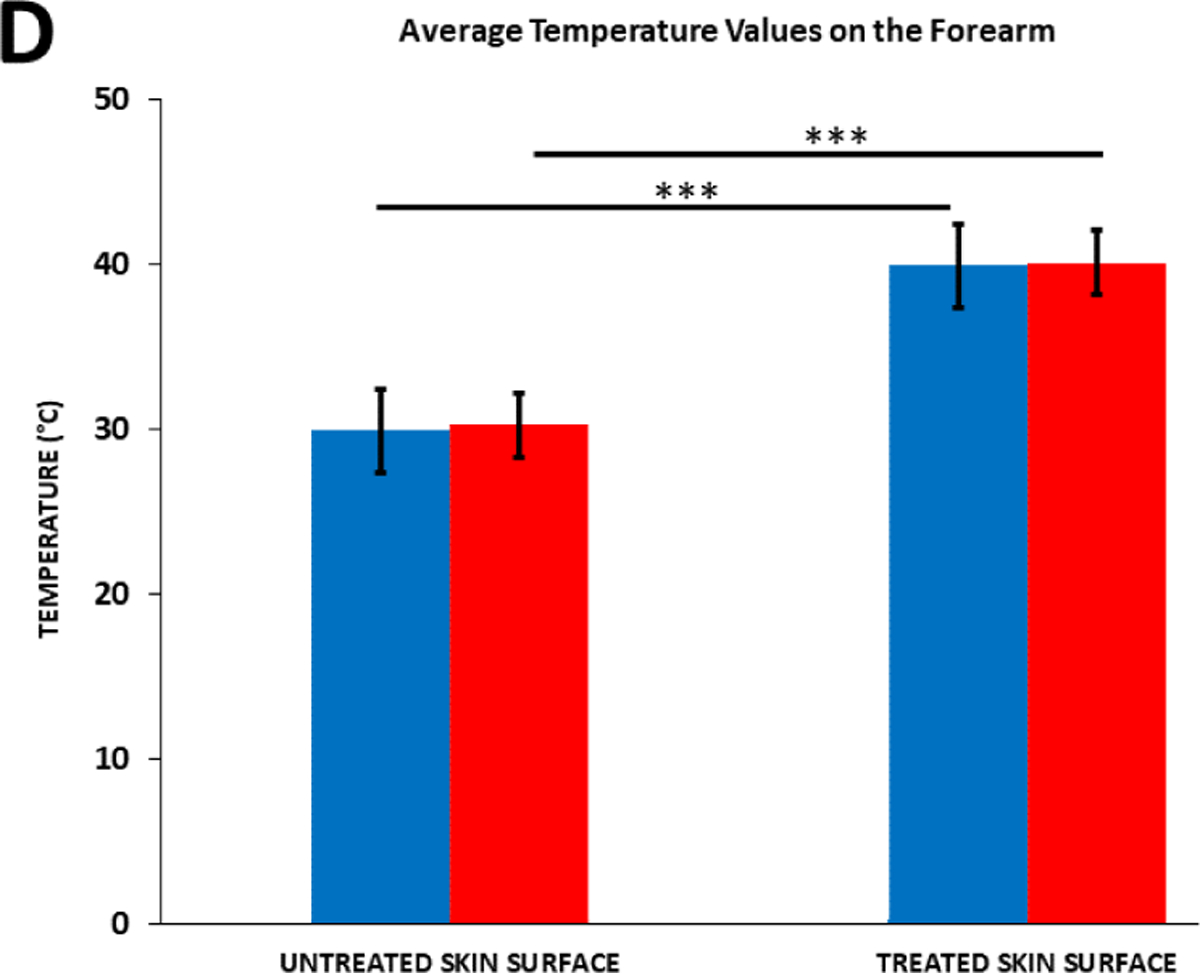
Average temperature values on the forearm before and after 4 hours of SAM stimulation. **Note:** (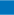) Ultrasound Gel; (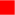) 2.5% Diclofenac Ultrasound Gel **Note:** *** = (p < 0.0001).

**Table 1:** Calculated values for Speed of Sound and Acoustic Impedance using equations (1) and (2), given density, and data values collected during intensity measurements (n=5).

	Standard ultrasound gel	2.5% Diclofenac gel	p-value (n=5)
Density (g/mL)	1.02	1.06	0.0001
Speed of Sound (m/s)	1512.64 ± 2.39	1513.24 ± 2.24	0.6944
Acoustic Impedance (Mpa.s/m^3^)	1.54 ± 0.002	1.60 ± 0.002	0.0001

**Table 2:** Ultrasound intensity at different depths stimulated with standard and 2.5% diclofenac ultrasound patches.

Depth (cm)	Intensity with standard gel (mW/cm^2^)	Intensity with 2.5% diclofenac gel (mW/cm^2^)
0	264.00 ± 1.12	264.00 ± 1.34
1	155.33 ± 5.37	166.04 ± 3.47
2	103.75 ± 7.92	106.50 ± 6.94
3	59.12 ± 6.33	70.32 ± 5.83
4	41.85 ± 4.51	48.33 ± 4.76
5	23.93 ± 1.87	34.97 ± 2.22
6	11.97 ± 1.32	26.84 ± 1.15
